# Diagnostic accuracy of strain imaging in predicting myocardial viability after an ST-elevation myocardial infarction

**DOI:** 10.1097/MD.0000000000019528

**Published:** 2020-05-08

**Authors:** Fathia Mghaieth Zghal, Selim Boudiche, Sofiane Haboubi, Henda Neji, Manel Ben Halima, Bassem Rekik, Mehdi Mechri, Sana Ouali, Saoussen Hantous, Mohamed Sami Mourali

**Affiliations:** a Service of Functional Exploration and Cardio Reanimation, Rabta University Hospital, Tunis, Tunisia; b Faculty of Medicine of Tunis, University Tunis El Manar, Tunis, Tunisia; c Service of Medical Imaging, Abderrahmane Mami University Hospital, Ariana, Tunisia.

**Keywords:** delayed enhancement cardiac magnetic resonance imaging, myocardial infarction, strain, sensitivity, specificity, viability

## Abstract

In the acute phase of ST-elevation myocardial infarction (STEMI) viability imaging techniques are not validated and/or not available.

This study aimed to evaluate the ability of strain parameters assessed in the acute phase of STEMI, to predict myocardial viability after revascularization.

Thirty-one STEMI patients whose culprit coronary artery was recanalized and in whom baseline echocardiogram showed an akinesia in the infarcted area, were prospectively included. Bidimensional left ventricular global longitudinal strain (GLS), and territorial longitudinal strain (TLS) in the territory of the infarct related artery were obtained within 24 hours from admission. Delayed enhancement (DE) cardiac magnetic resonance imaging (CMR) was used as a reference test to assess post-revascularization myocardial viability. DE-CMR was performed 3 months after percutaneous coronary intervention. According to myocardial viability, patients were divided into 2 groups; CMR viable myocardium patients with more than half of infarcted segments having a DE <50% (group V) and CMR nonviable myocardium patients with half or more of the infarcted segments having a DE >50% (group NV).

GLS and TLS were lower in group V compared to group NV (respectively: −14.4% ± 2.9% vs −10.9% ± 2.4%, *P* = .002 and −11.0 ± 4.1 vs −3.2 ± 3.1, *P* = .001). GLS was correlated with DE-CMR (*r* = 0.54, *P* = .002) and a cut off value of −13.9% for GLS predicted viability with 86% sensitivity (Se) and 78% specificity (Sp). TLS showed the strongest correlation with DE-CMR (*r* = 0.69, *P* < .001). A cut off value of −9.4% for TLS yielded a Se of 78% and a Sp of 95% to predict myocardial viability.

GLS and TLS measured in the acute phase of STEMI predicted myocardial viability assessed by 3 months DE-CMR. They are prognostic indicators and they can be used to guide the priority and usefulness of percutaneous coronary intervention in these patients.

## 1 Introduction

In ST-elevation myocardial infarction (STEMI) the main treatment consists in immediate coronary artery recanalization. This strategy aims to restore the coronary flow and myocardial perfusion and, consequently, to recover myocardial contractile function. For prognostic and therapeutic purposes, it is important to predict whether myocardium with severe kinetic dysfunction in the acute phase of STEMI is irreversibly necrotic or reversibly stunned.

Conventional echocardiography with visual assessment of regional and global myocardial function is not able to predict viability.^[1,2]^ Delayed enhancement cardiac magnetic resonance imaging (DE-CMR) is currently the criterion standard for myocardial viability assessment. Apart from the problem of its immediate unavailability, DE-CMR was mainly validated in chronic ischemic disease,^[3]^ and its validity in the acute phase of STEMI is not established.

Bidimensional (2D) strain is a key predictor of left ventricular (LV) remodeling and prognosis after STEMI. 2D strain also showed a good correlation with DE-CMR in chronic ischemic disease for viability assessment.^[4,5]^ It has been recently suggested that 2D strain in acute coronary syndrome can predict later myocardial viability.

Our aim was to evaluate the ability of 2D global longitudinal strain (GLS) of the LV and territorial longitudinal strain (TLS) measured in the acute phase of STEMI, to predict later myocardial viability after revascularization assessed by DE-CMR performed at 3 months.

## 2 Population and method

The study was prospective, monocentric conducted in consecutive patients admitted to the intensive care unit of our teaching hospital between January and April 2019. It was approved by the institutional ethics committee.

### 2.1 Patients’ selection

Patients were enrolled after they provided an informed consent. We included adult patients (≥18 years) hospitalized for a first STEMI episode whose echocardiography showed an akinetic myocardial area and whose culprit coronary artery was recanalized. Exclusion criteria were previous (known or identified by baseline echocardiography) STEMI, non-STEMI, or chronic ischemic myocardial disease, the absence of akinesia in the infarcted territory, the absence of recanalization of the culprit artery, a complete left bundle branch block, an atrial fibrillation, and an implanted pacemaker or automated defibrillator device.

### 2.2 Echocardiography protocol

An echocardiography was performed by the same experienced operator in all patients within 24 hours of admission. Echocardiograms were realized on a General Electric Vivid E9 machine equipped with a 2.5 to 5 MHz variable frequency phased array transducer. Cardiac chambers’ quantifications followed the American Society of Echocardiography and the European Association of cardiovascular imaging guidelines.^[6]^ Parietal kinetic assessment was performed visually in apical 4, 2, and 3 chamber views and parasternal long-axis and short-axis views. We used a 17 segments LV model and a score was attributed to each analyzed segment; 1 = normal, 2 = hypokinetic, 3 = akinetic and 4 = dyskinetic. The wall motion score index (WMSI) was calculated as the average score of all analyzed segments. Three regional WMSIs were calculated by averaging the scores of segments in each of the 3 coronary artery distribution areas; the left anterior descending (LAD) artery area, the circumflex artery (Cx) area and the right coronary artery (RCA) area.

Two-dimensional strain analysis was performed on gray scale acquisitions in 4, 2, and 3 chamber views, centered on the LV with an image rate between 60 and 80 per second. An automated function imaging software, available on the echocardiography machine, allowed the measurement of segmental and GLS. TLS was calculated by averaging manually strain values of segments related to the same coronary artery. By analogy to regional WMSI we obtained three territorial strain values; TLS LAD, TLS Cx and TLS RCA.

### 2.3 Delayed enhancement cardiac magnetic resonance imaging protocol

CMR images analysis were performed concomitantly by two experienced radiologists who were blind to echocardiographic findings. Baseline magnetic resonance images were acquired using a Philips Ingenia 1.5 T system with Intellispace 6 console. For DE-CMR analysis, a 17-segment LV model was used. The protocol included short-axis and 4-chamber cine acquisitions, black blood sequences weighted in T2 and/or short tau inversion recovery in short-axis, first-pass dynamic perfusion sequence or early gadolinium enhancement (within the first 1–3 minutes after contrast infusion) to look for a microvascular obstruction indicating a no reflow, and late gadolinium enhancement (15 min after contrast infusion) using phase-sensitive inversion recovery sequences technique for the determination of transmurality.

The cut off value of 50% of DE was considered to define segmental myocardial viability.^[7]^ A myocardial territory was qualified as viable when more than half of its segments had a DE ≤50% and nonviable when half or more of its segments had a DE >50%.

### 2.4 Initial investigation and follow-up of patients

For each patient data were collected prospectively, including clinical, echocardiographic, and angiographic data, as well as percutaneous coronary intervention (PCI) course were recorded. Intrahospital complications were reported. Major cardiovascular events included death, resuscitated cardiac arrest, cardiogenic shock, myocardial infarction, and stroke. PCI results were assessed by the thrombolysis in myocardial infarction score in the culprit vessel.^[8]^ The echocardiographic examination, as described above, was performed in all patients in the acute phase and repeated at 3 months. All patients had a 3 months clinical follow-up. DE-CMR was performed at 3 months after STEMI occurrence.

Substudy groups, group V (viable) and group NV (nonviable), were defined according to viability assessment of the myocardial territory related to the culprit coronary artery by DE-CMR.

### 2.5 Statistical analysis

A statistical software SPSS 22.0 was used. Categorical variables were expressed in numbers and percentages. Quantitative variables were expressed in means and standard deviations. Comparisons of quantitative variables between the 2 groups were made using the Mann-Whitney *U* test, and for correlation assessment we calculated Spearman coefficient. A *P* value <.05 was considered statistically significant. Sensitivity (Se) and specificity (Sp) for prediction of myocardial viability was determined by performing receiver operating characteristic curve analysis. The value of the variable with the best Se-Sp couple was chosen as the cut-off value, 95% confidence intervals of its Se and Sp were calculated.

## 3 Results

### 3.1 General characteristics of the population

**Figure 1:**
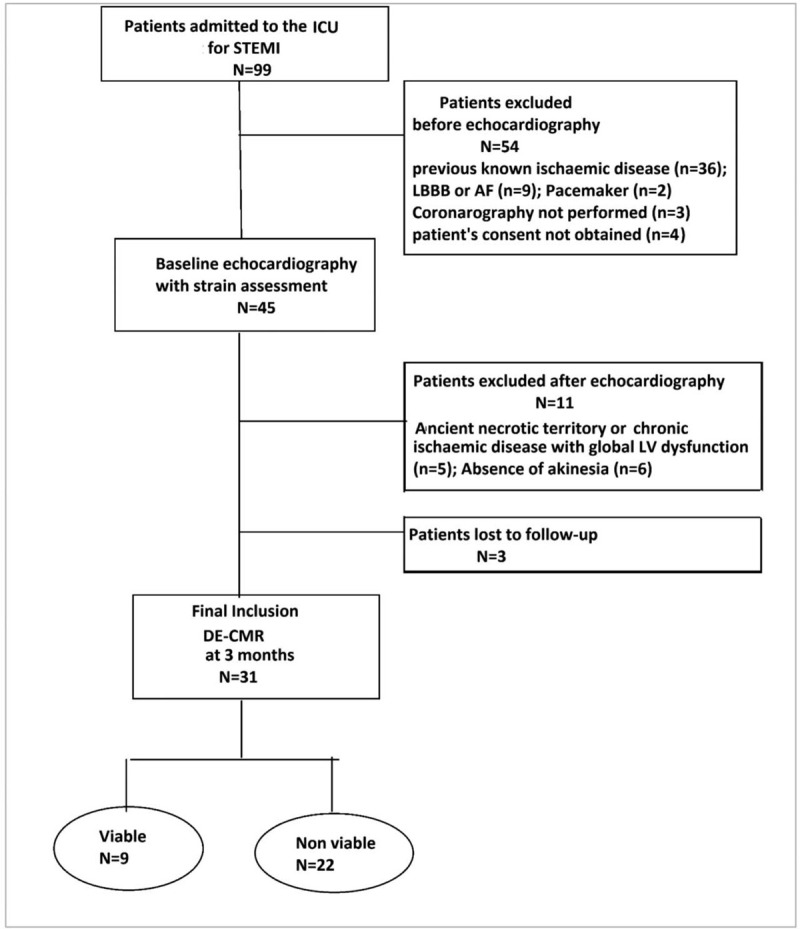
Design of the study. AF = atrial fibrillation, ICU = intensive care unit, LBBB = left bundle branch block, LV = left ventricle, STEMI = ST-elevation myocardial infarction.

Figure 1 represents the design of the study. Our cohort consisted in 31 patients (87% males) enrolled between January 2019 and April 2019, in our ICU, with a 3 months follow-up for each patient. Mean age was 59.2 ± 10.1 years (41–84 years). Cigarette smoking was the most frequent risk factor found in 74% of patients. The LAD was the culprit vessel in 22 patients (71%), the Cx in 3 patients (10%) and the RCA in 6 patients (19%). Twenty-nine patients had a PCI in the culprit artery. Primary PCI was performed in 18 patients (58%), rescue PCI in 4 patients (13%) and elective PCI in 7 patients (22%). Only 1 patient underwent a coronary artery bypass grafting 120 hours after a successful fibrinolysis, and 1 patient had a permeable coronary artery at angiography (thrombolysis in myocardial infarction flow 3) without significant stenosis, he did not necessitate an angioplasty. Intrahospital major cardiovascular event occurred in 5 patients (16%) all of whom belonged to the NV group.

Table 1 shows the general and angiographic characteristics of the population, there was no significant difference regarding these characteristics between the 2 groups.

**Table 1:**
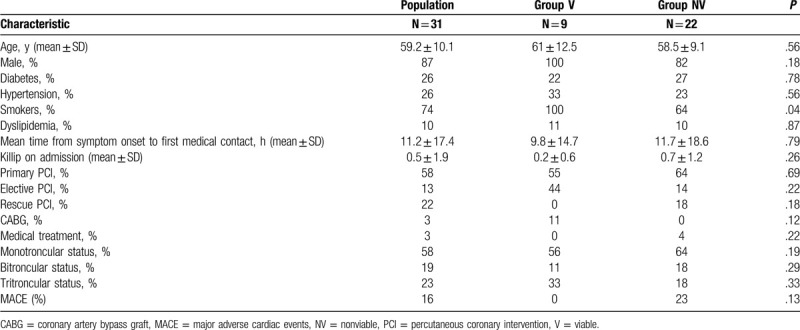
General and angiographic characteristics.

### 3.2 Cardiac magnetic resonance characteristics

Among 527 segments evaluated, 94 (17.83%) were nonviable. Nine patients (29%) had a viable myocardium in the territory of the culprit artery (substudy group V) and 22 patients (71%) had a nonviable infarcted myocardial territory (substudy group NV) (Table 2). At 3 months, CMR LVEF was higher in group V (48.7 ± 7.9 vs 38.7 ± 8.7, *P* = .008). The early gadolinium enhancement images were assessed in 27 patients, microvascular obstruction (no reflow) was only found in group NV, in 15 patients (*P* = .001).

**Table 2:**
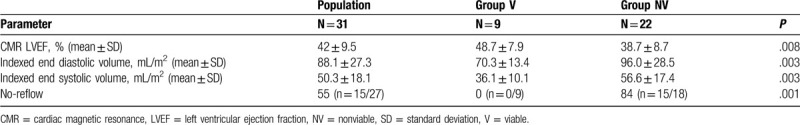
Cardiac magnetic resonance imaging parameters.

### 3.3 Echocardiographic characteristics

Table 3 summarizes echocardiographic characteristics. Among 527 segments evaluated visually, 112 (21.25%) were akinetic. At the acute phase of STEMI, mean LVEF was 44.9% ± 9.0% and was higher in group V than in group NV (50.7% ± 6.7% vs 42.5% ± 8.9%, *P* = .02). At 3 months, LVEF remained higher in group V (*P* = .001). Regional WMSI in the culprit artery territory was lower in group V at baseline and at 3 months. Segmental strain was achieved in 518 (98.3%) segments. Global and Territorial strain measures were obtained in all patients. Strain parameters were better in group V at baseline echocardiography (−14.4% ± 2.9% vs −10.9% ± 2.4%, *P* = .002) for GLS and (−11.0% ± 4.1% vs −3.2% ± 3.1%, *P* < .001) for TLS. These 2 parameters showed a greater improvement at 3 months in group V, which was for GLS (−3.3% ± 1.9% vs −1.6% ± 1.3%, *P* = .001) and for TLS (−6.1% ± 3.7% vs 4.2% ± 3.3%, *P* = .001).

**Table 3:**
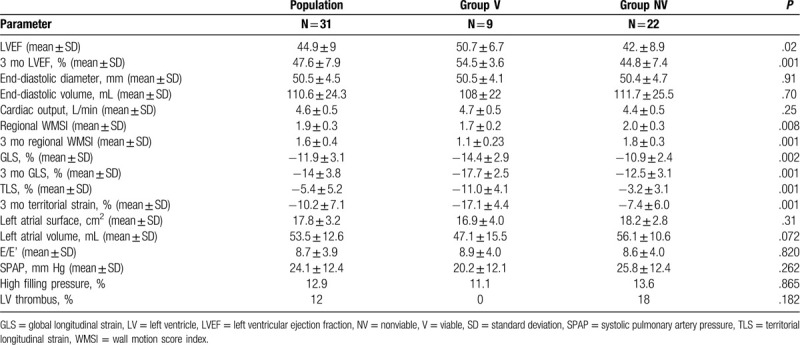
Echocardiographic study.

As shown in Table 4, baseline LVEF was not predictive of viability and had no significant correlation with DE-CMR (*P* = .19). Similarly, regional WMSI in the culprit artery territory was not correlated with DE-CMR (*P* = .08).

**Table 4:**
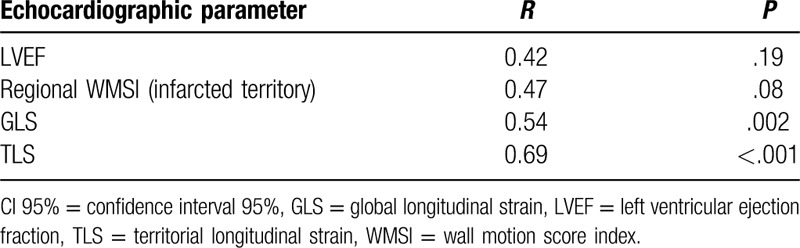
Correlation between echocardiographic parameters and delayed enhancement in cardiac magnetic resonance imaging.

A significant correlation was found between GLS and DE-CMR (*r* = .54, *P* = .002). A cut off value of −13.9% for GLS predicted viability with 86% Se and 78% Sp (Table 5).

**Table 5:**
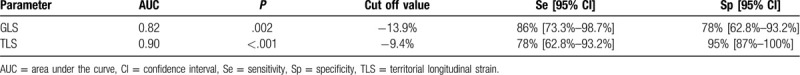
Sensitivity and specificity of global and territorial strain to predict viability.

TLS showed the best correlation with DE-CMR (*r* = 0.690, *P* < .001). A cut off value of −9.4% for TLS predicted viability with 78% Se and 95% Sp, whereas a cut off value of −5.5% for TLS predicted nonviability with 89% Se and 77% Sp. Extreme values of TLS >−2.5% predicted nonviability with 100% Sp.

In our population, 18 patients had a TLS >−5.5%, among them only 1 patient, with a TLS = −3%, had a viable myocardium.

## 4 Discussion

In our study, we showed in a prospective cohort of 31 patients hospitalized for a first STEMI episode that GLS and TLS measured at the acute phase allowed an accurate prediction of myocardial viability and had a good correlation with DE-CMR performed at 3 months after STEMI occurrence. Thus, strain parameters could differentiate myocardium with reversible dysfunction that deserves revascularization from permanently damaged myocardium. A TLS <−9.4% predicted viability with a high specificity (95%).

### 4.1 Diagnosis of myocardial viability by DE-CMR and its limitations

Among CMR techniques of viability assessment in patients with chronic LV dysfunction due to coronary artery disease, DE-CMR had a high sensitivity (95%) and a high negative predictive value,^[9]^ and it is currently used as a criterion standard. The use of DE-CMR for viability assessment was on the contrary, very limited during the acute phase of STEMI. In this setting CMR is rarely available and was not validated due to frequent false positives. Indeed, parietal edema in the acute phase of STEMI led to an overestimation of the infarct size by including parts of the area at risk.^[10]^ Gadolinium retention was caused by to a change in extracellular volume due to the rupture of cardiomyocyte membrane and to a slowing of its clearance.^[11]^ All these factors skewed the assessment of myocardial viability and led to inappropriate therapeutic strategies. In our study, DE-CMR was performed 3 months after the STEMI episode to ensure an accurate assessment of myocardial viability.

### 4.2 Viability and left ventricular functional recovery

Early recognition of viable myocardium has clinical relevance, because only viable segments have the potential of functional recovery with improved clinical outcome.^[12,13]^ Sciagra et al^[12]^ evaluated 48 patients with STEMI who were treated with primary PCI. The assessment of the viability of the infarcted zone with low-dose dobutamine echocardiography at 3 days allowed prediction of LV function improvement at 6 months. In another cohort of 300 STEMI patients, it was demonstrated that viability assessed by low-dose dobutamine echocardiography performed in the early phase, was associated with a better 9 months survival.^[13]^

### 4.3 Left ventricular ejection fraction, wall motion score index, and viability prediction

LV function assessed by 2D echocardiography is among the most important determinants of prognosis in STEMI patients and it contributes to therapeutic decisions.^[14]^ Several studies demonstrated that, in acute coronary syndromes, LV dysfunction is potentially reversible, related to myocardial stunning, myocardial hibernation, or a combination of these 2 processes; therefore, LV function may improve after revascularization confirming myocardial viability.^[15]^ If this reversibility is predicted by baseline LVEF is however questionable. In our study, LVEF was not significantly correlated with DE-CMR (*P* = .19). This result corroborated those of previous studies that did not found a significant relationship between baseline LVEF and 1-year functional recovery.^[16]^

Concerning WMSI, it was found in some studies to be more accurate than LVEF for morbidity and mortality prediction after an ACS.^[17]^ In our study, we did not find a significant correlation between WMSI and DE-CMR viability (*P* = .08). It was previously reported in patients with a first anterior STEMI who were revascularized, that a lower WMSI was a predictor of contractile recovery,^[18]^ but this result was assessed by an echocardiographic follow-up without a reference method for viability assessment.

### 4.4 Longitudinal strain and viability assessment

Myocardial deformation estimation by either tissue Doppler or speckle tracking 2D strain predicted accurately viability in patients with chronic ischemic LV dysfunction^[4,5]^ but few studies evaluated the usefulness of 2D strain to predict viability when assessed at the acute phase of STEMI, even if the prognostic value of this parameter was widely demonstrated. In fact, GLS was a strong predictor of LV remodeling and adverse events such as congestive heart failure and death.^[19]^ In chronic ischemic myocardial disease, a cut off value of −13% for TLS, performed 9 months after a first STEMI, identified a transmural infarction assessed by DE-CMR with 80% Se and 83% Sp.^[20]^

In an acute setting, in STEMI patients, we found a correlation between baseline GLS and 3 months DE-CMR and we identified a cut off value of −13.9% to predict viability with 86% Se and 78% Sp, and we found that TLS was a better parameter for viability prediction, a cut off value of −9.4% for TLS predicted viability with a Se of 78% and a Sp of 95%.

Eek et al^[21]^ found also a good correlation between infarct size and GLS in a population of 61 NSTEMI in comparison with 9 ± 3 months DE-CMR and a cut off value of −13.8%, close to our finding, identified patients with a transmural infarction. In a meta-analysis published in 2017 that pooled 11 prospective randomized studies led in patients after a first myocardial infarction (N = 765) either STEMI (6 studies) or non-STEMI (2 studies) or both (3 studies), it was reported a significant correlation between 2D GLS and DE-CMR (*r* = 0.70; 95% confidence interval: 0.64, 0.74). GLS predicted an infarct size >12% with a Se of 77% and a Sp of 86%.^[22]^ It should be noticed that in this meta-analysis, only in 3 studies^[23–25]^ that enrolled respectively 30,^[23]^ 39,^[24]^ and 44^[25]^ patients, strain assessment was performed in the early phase of STEMI by Doppler imaging^[23]^ or by speckle tracking imaging.^[24,25]^ The other studies either did not include STEMI patients or reported late assessment of strain, beyond the acute phase. In the 2 more recent and largest studies that used speckle tracking 2D strain,^[24,25]^ all patients had a primary PCI, and strain was assessed after revascularization. The presence of an akinesia at baseline echocardiogram was not a required inclusion criterium. This can explain the different strain cut off values identified and the more frequent nonviable myocardium in our series. Indeed, our study not only targeted a prognostic assessment but also suggested a guiding of the therapeutic strategy based on strain in patients with borderline indications to PCI. Furthermore, if these 3 studies reported a significant correlation between GLS and 3 or 9 months DE-CMR, in agreement to our findings, none of them reported results with TLS, which had a stronger correlation with DE-CMR in our study.

For Cimino et al^[26]^ GLS provided also an accurate assessment of transmurality, but corroborating our results, they found that the best correlation with DE-CMR was obtained with the regional strain and a cut off value of −12% for TLS identified enhanced areas with 82% Se and 78% Sp.

Recently, a study led in 100 patients with anterior STEMI treated with primary PCI demonstrated that baseline GLS could predict infarct size.^[27]^ The cut off value of −13% for GLS, identified large infarct size with 66.7% Se and 88.4% Sp. The authors found also that for TLS, a cut off value of −9.6% predicted viability with a 94% Se and 86% Sp. These results are close and perfectly corroborate our findings.

### 4.5 Clinical implication

This prospective cohort study was in favor of a good correlation between early 2D strain and later DE-CMR in STEMI patients. TLS was a strong predictor of myocardial viability. These findings can guide the revascularization strategy in STEMI patients along with a global clinical assessment, this can be important in frail elderly patients, patients with acute renal failure and late revascularizations. Particularly for these patients, we suggest to:

- Target by revascularization patients with very likely myocardial viability (TLS <−9.4%)
- Discuss revascularization on a case-by-case basis for borderline patients with a TLS between −9.4% and −5.5%.
- Avoid unnecessary revascularizations when viability is very unlikely (TLS >−5.5%), and thus avoid potential iatrogenic complications and unnecessary expenditure.

### 4.6 Study limitations

First, the number of patients included was not high but despite this our results were statistically significant. Moreover, we did not include patients with ventricular stimulation and left bundle branch block to avoid misinterpretations of strain and visual kinetic assessment. These patients, however, could represent a specific target population of such studies, because STEMI diagnosis and management are more difficult in them. Finally, the calculation of the TLS was based on a theoretical coronary distribution and we did not consider anatomical patients’ particularities, especially the coronary dominance.

## 5 Conclusion

In the acute phase of STEMI, 2D strain parameters GLS and TLS can accurately predict post-revascularization myocardial viability, which could help prognosis assessment and therapeutic strategy guiding.

## Acknowledgment

The authors acknowledge Pr Zouari Bechir for verifying all the statistical analysis.

## Author contributions

Dr Mghaieth Zghal was the echocardiography operator, main investigator of the study and main author of the manuscript. Dr Boudiche, Dr Haboubi and Dr Mechri gathered clinical and angiographic data and performed statistics, Dr Ben Halima, Dr Rekik and Dr Ouali performed bibliography research, contributed to the writing of the parts of the manuscript, and helped in language corrections they also gave deep revision remarks. Dr Hantous and Dr Neji performed CMR, and wrote CMR protocol, results ans discussion. Dr Mourali validated the protocol of the research, and gave overall deep revision of the manuscript.
